# 1,1,1,3,3,3-Hexafluoro-2,2-bis­[4-(4-nitro­phen­oxy)phen­yl]propane

**DOI:** 10.1107/S1600536808022101

**Published:** 2008-07-19

**Authors:** H. Nawaz, Zareen Akhter, Michael Bolte, M .S. Butt, Humaira M. Siddiqi

**Affiliations:** a Department of Chemistry, Quaid-I-Azam University, Islamabad 45320, Pakistan; b Institut für Anorganische Chemie, J. W. Goethe-Universität Frankfurt, Max-von-Laue-Strasse 7, 60438 Frankfurt/Main, Germany

## Abstract

In the title compound, C_27_H_16_F_6_N_2_O_6_, the nitro groups are almost coplanar with the aromatic rings to which they are attached [dihedral angles = 3.5 (5) and 6.2 (3)°]. The dihedral angles between adjacent aromatic rings are 78.07 (8) and 71.11 (8)° for nitro­phen­yl/phenyl and 69.50 (8)° for phen­yl/phenyl. An inter­molecular C—H⋯π inter­action seems to be effective in the stabilization of the structure.

## Related literature

For related literature, see: Liaw *et al.* (2005 ▶); Yang *et al.* (2003 ▶); Miyagawa *et al.* (2003 ▶); Leu *et al.* (2003 ▶); Zhou *et al.* (2001 ▶.
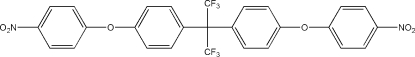


## Experimental

### 

#### Crystal data

C_27_H_16_F_6_N_2_O_6_
*M* *_r_* = 578.42
Monoclinic, 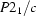

*a* = 25.523 (3) Å
*b* = 10.5530 (12) Å
*c* = 9.3869 (8) Å
β = 98.248 (8)°
*V* = 2502.2 (5) Å^3^
*Z* = 4
Mo *K*α radiation
μ = 0.14 mm^−1^
*T* = 173 (2) K
0.23 × 0.10 × 0.10 mm

#### Data collection

Stoe IPDSII two-circle diffractometer
Absorption correction: none
13020 measured reflections
4653 independent reflections
2651 reflections with *I* > 2σ(*I*)
*R*_int_ = 0.076

#### Refinement

*R*[*F* ^2^ > 2σ(*F* ^2^)] = 0.044
*wR*(*F* ^2^) = 0.092
*S* = 0.91
4653 reflections
371 parameters
H-atom parameters constrained
Δρ_max_ = 0.20 e Å^−3^
Δρ_min_ = −0.21 e Å^−3^

### 

Data collection: *X-AREA* (Stoe & Cie, 2001 ▶); cell refinement: *X-AREA*; data reduction: *X-AREA*; program(s) used to solve structure: *SHELXS97* (Sheldrick, 2008 ▶); program(s) used to refine structure: *SHELXL97* (Sheldrick, 2008 ▶); molecular graphics: *XP* in *SHELXTL-Plus* (Sheldrick, 2008 ▶); software used to prepare material for publication: *SHELXL97*.

## Supplementary Material

Crystal structure: contains datablocks I, global. DOI: 10.1107/S1600536808022101/bq2089sup1.cif

Structure factors: contains datablocks I. DOI: 10.1107/S1600536808022101/bq2089Isup2.hkl

Additional supplementary materials:  crystallographic information; 3D view; checkCIF report

## Figures and Tables

**Table 1:** Hydrogen-bond geometry (Å, °)

*D*—H⋯*A*	*D*—H	H⋯*A*	*D*⋯*A*	*D*—H⋯*A*
C46—H46⋯*Cg*1^i^	0.95	3.04	3.710	129

Symmetry code: (i) 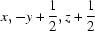. *Cg*1 is the centroid of the C31–C36 ring.

**Fig. 1.**
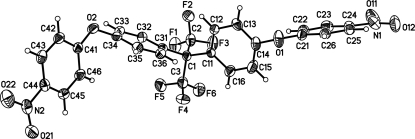
Perspective view of the title compound with the atom numbering; displacement ellipsoids are at the 50% probability level.

## supplementary crystallographic information

### Comment

Aromatic polyimides have many useful properties, such as high glass transition temperature, excellent dimensional stability, low dielectric constant and outstanding thermal stability (Liaw *et al.* 2005). Polyimides also have many commercial applications but one of the problems with the polyimides is their poor solubility (Yang *et al.* 2003). Many efforts have been made to increase the solubility (Miyagawa *et al.* 2003) and processability of the polyimides. The common strategy that enhances the solubility is the introduction of flexible linkages, bulky substituents (Leu *et al.* 2003) and structurally unsymmetrical segment (Zhou *et al.* 2001) in to the polymer backbone. The present compound is the starting material for such types of polymers.

Geometric parameters of (I) are in the usual ranges. The nitro groups are almost coplanar with the aromatic rings to which they are attached [dihedral angles: 3.5 (5)128.52 and 6.2 (3)°], (Figure 1.). The dihedral angles between the adjacent aromatic rings are 78.07 (8)° and 71.11 (8)° for nitrophenyl/phenyl and 69.50 (8)° for phenyl/phenyl. The C46···H46-Cg1 intermolecular interaction seems to be effective in the stabilization of the structure. Cg1 is the center of the ring (C31-C36) at the symmetry of x, 1/2-y, 1/2+z (Table 1.)

### Experimental

A mixture containing 2 g (5.94 mmol) of 4,4'-(hexafluoroisopropylidene)-diphenol, 2.25 g (11.88 mmol) of anhydrous potassium carbonate, and 1.87 g (11.88 mmoles) of *p*-nitrochlorobenzene, in 70 mL of DMF was heated at 120° C for 18 h in nitrogen atmosphere. After cooling to room temperature, the reaction mixture was poured into 500 mL of water to form precipitates and washed thoroughly with water and then collected by filtration. The crude product was recrystallized from ethanol. m.p.352 K.

### Refinement

H atoms could be located by difference Fourier synthesis. They were refined with fixed individual displacement parameters [U(H) = 1.2 *U*_eq_(C)] using a riding model with C—H = 0.95 Å.

### Crystal data

**Table:**

C_27_H_16_F_6_N_2_O_6_	*F*_000_ = 1176
*M**_r_* = 578.42	*D*_x_ = 1.535 Mg m^−^^3^
Monoclinic, *P*2_1_/*c*	Mo *K*α radiation λ = 0.71073 Å
Hall symbol: -P 2ybc	Cell parameters from 6504 reflections
*a* = 25.523 (3) Å	θ = 3.5–25.6º
*b* = 10.5530 (12) Å	µ = 0.14 mm^−^^1^
*c* = 9.3869 (8) Å	*T* = 173 (2) K
β = 98.248 (8)º	Rod, colourless
*V* = 2502.2 (5) Å^3^	0.23 × 0.10 × 0.10 mm
*Z* = 4	

### Data collection

**Table:**

Stoe IPDSII two-circle diffractometer	2651 reflections with *I* > 2σ(*I*)
Radiation source: fine-focus sealed tube	*R*_int_ = 0.076
Monochromator: graphite	θ_max_ = 25.6º
*T* = 173(2) K	θ_min_ = 3.5º
ω scans	*h* = −30→31
Absorption correction: none	*k* = −12→12
13020 measured reflections	*l* = −11→9
4653 independent reflections	

### Refinement

**Table:**

Refinement on *F*^2^	Hydrogen site location: inferred from neighbouring sites
Least-squares matrix: full	H-atom parameters constrained
*R*[*F*^2^ > 2σ(*F*^2^)] = 0.044	*w* = 1/[σ^2^(*F*_o_^2^) + (0.0358*P*)^2^] where *P* = (*F*_o_^2^ + 2*F*_c_^2^)/3
*wR*(*F*^2^) = 0.092	(Δ/σ)_max_ < 0.001
*S* = 0.91	Δρ_max_ = 0.20 e Å^−^^3^
4653 reflections	Δρ_min_ = −0.21 e Å^−^^3^
371 parameters	Extinction correction: SHELXL97 (Sheldrick, 2008), Fc^*^=kFc[1+0.001xFc^2^λ^3^/sin(2θ)]^-1/4^
Primary atom site location: structure-invariant direct methods	Extinction coefficient: 0.0055 (6)
Secondary atom site location: difference Fourier map	

### Special details

**Table:**

Geometry. All e.s.d.'s (except the e.s.d. in the dihedral angle between two l.s. planes) are estimated using the full covariance matrix. The cell e.s.d.'s are taken into account individually in the estimation of e.s.d.'s in distances, angles and torsion angles; correlations between e.s.d.'s in cell parameters are only used when they are defined by crystal symmetry. An approximate (isotropic) treatment of cell e.s.d.'s is used for estimating e.s.d.'s involving l.s. planes.
Refinement. Refinement of *F*^2^ against ALL reflections. The weighted *R*-factor *wR* and goodness of fit *S* are based on *F*^2^, conventional *R*-factors *R* are based on *F*, with *F* set to zero for negative *F*^2^. The threshold expression of *F*^2^ > σ(*F*^2^) is used only for calculating *R*-factors(gt) *etc*. and is not relevant to the choice of reflections for refinement. *R*-factors based on *F*^2^ are statistically about twice as large as those based on *F*, and *R*- factors based on ALL data will be even larger.

### Fractional atomic coordinates and isotropic or equivalent isotropic displacement parameters (Å^2^)

**Table:**

	*x*	*y*	*z*	*U*_iso_*/*U*_eq_	
F1	0.22322 (6)	0.68466 (15)	0.12465 (17)	0.0501 (5)	
F2	0.20394 (6)	0.68591 (13)	0.33963 (17)	0.0410 (4)	
F3	0.28426 (6)	0.71700 (14)	0.30377 (18)	0.0506 (5)	
F4	0.30099 (6)	0.34542 (16)	0.17262 (18)	0.0535 (5)	
F5	0.25025 (6)	0.46271 (18)	0.02529 (16)	0.0551 (5)	
F6	0.32280 (6)	0.54001 (17)	0.13798 (17)	0.0552 (5)	
O1	0.36452 (7)	0.43650 (18)	0.8434 (2)	0.0482 (5)	
O2	0.06515 (6)	0.19665 (15)	0.2107 (2)	0.0379 (5)	
N1	0.50826 (10)	0.7761 (3)	1.1374 (3)	0.0511 (7)	
O11	0.51204 (11)	0.8833 (2)	1.0897 (3)	0.0842 (9)	
O12	0.53558 (8)	0.7378 (2)	1.2468 (2)	0.0641 (7)	
N2	0.06342 (9)	−0.2973 (2)	0.0006 (2)	0.0366 (5)	
O21	0.09940 (8)	−0.33014 (17)	−0.0658 (2)	0.0467 (5)	
O22	0.02828 (8)	−0.36920 (18)	0.0283 (2)	0.0544 (6)	
C1	0.25201 (9)	0.5041 (2)	0.2760 (2)	0.0282 (5)	
C2	0.24075 (10)	0.6489 (2)	0.2604 (3)	0.0352 (6)	
C3	0.28207 (10)	0.4635 (3)	0.1534 (3)	0.0414 (7)	
C11	0.28502 (9)	0.4859 (2)	0.4265 (3)	0.0264 (5)	
C12	0.25853 (9)	0.4963 (2)	0.5458 (3)	0.0285 (6)	
H12	0.2216	0.5127	0.5325	0.034*	
C13	0.28577 (10)	0.4830 (2)	0.6843 (3)	0.0340 (6)	
H13	0.2676	0.4903	0.7655	0.041*	
C14	0.33916 (10)	0.4592 (2)	0.7024 (3)	0.0358 (6)	
C15	0.36642 (10)	0.4496 (3)	0.5880 (3)	0.0449 (7)	
H15	0.4034	0.4337	0.6030	0.054*	
C16	0.33941 (9)	0.4632 (3)	0.4487 (3)	0.0383 (7)	
H16	0.3582	0.4570	0.3686	0.046*	
C21	0.39889 (9)	0.5259 (2)	0.9095 (3)	0.0328 (6)	
C22	0.40440 (10)	0.6473 (3)	0.8560 (3)	0.0379 (7)	
H22	0.3842	0.6729	0.7678	0.045*	
C23	0.43979 (10)	0.7308 (3)	0.9330 (3)	0.0400 (7)	
H23	0.4439	0.8143	0.8986	0.048*	
C24	0.46891 (10)	0.6905 (3)	1.0604 (3)	0.0353 (6)	
C25	0.46318 (10)	0.5707 (3)	1.1147 (3)	0.0355 (6)	
H25	0.4834	0.5455	1.2030	0.043*	
C26	0.42775 (9)	0.4881 (3)	1.0393 (3)	0.0343 (6)	
H26	0.4231	0.4056	1.0759	0.041*	
C31	0.20057 (9)	0.4246 (2)	0.2604 (2)	0.0253 (5)	
C32	0.15356 (9)	0.4636 (2)	0.1781 (3)	0.0287 (6)	
H32	0.1520	0.5445	0.1332	0.034*	
C33	0.10890 (9)	0.3865 (2)	0.1602 (3)	0.0312 (6)	
H33	0.0770	0.4149	0.1049	0.037*	
C34	0.11153 (9)	0.2691 (2)	0.2234 (3)	0.0301 (6)	
C35	0.15786 (9)	0.2261 (2)	0.3063 (3)	0.0320 (6)	
H35	0.1591	0.1449	0.3504	0.038*	
C36	0.20206 (9)	0.3040 (2)	0.3230 (3)	0.0309 (6)	
H36	0.2340	0.2750	0.3780	0.037*	
C41	0.06640 (9)	0.0749 (2)	0.1551 (3)	0.0282 (6)	
C42	0.02822 (9)	−0.0074 (2)	0.1911 (3)	0.0305 (6)	
H42	0.0035	0.0201	0.2513	0.037*	
C43	0.02635 (9)	−0.1297 (2)	0.1388 (3)	0.0323 (6)	
H43	0.0005	−0.1878	0.1621	0.039*	
C44	0.06299 (9)	−0.1656 (2)	0.0517 (3)	0.0288 (6)	
C45	0.10087 (9)	−0.0837 (2)	0.0139 (3)	0.0311 (6)	
H45	0.1255	−0.1112	−0.0464	0.037*	
C46	0.10214 (9)	0.0389 (2)	0.0653 (3)	0.0316 (6)	
H46	0.1273	0.0978	0.0393	0.038*	

### Atomic displacement parameters (Å^2^)

**Table:**

	*U*^11^	*U*^22^	*U*^33^	*U*^12^	*U*^13^	*U*^23^
F1	0.0590 (10)	0.0512 (10)	0.0374 (10)	−0.0124 (8)	−0.0025 (8)	0.0207 (8)
F2	0.0454 (9)	0.0307 (8)	0.0468 (10)	0.0004 (7)	0.0066 (7)	−0.0023 (7)
F3	0.0459 (9)	0.0431 (9)	0.0595 (11)	−0.0219 (8)	−0.0034 (8)	0.0081 (8)
F4	0.0509 (10)	0.0638 (12)	0.0482 (11)	0.0104 (9)	0.0159 (8)	−0.0167 (9)
F5	0.0504 (9)	0.0941 (13)	0.0215 (8)	−0.0100 (9)	0.0076 (7)	−0.0080 (9)
F6	0.0388 (9)	0.0910 (13)	0.0392 (9)	−0.0180 (9)	0.0170 (7)	−0.0004 (9)
O1	0.0504 (11)	0.0580 (13)	0.0309 (11)	−0.0141 (10)	−0.0124 (9)	0.0125 (9)
O2	0.0260 (9)	0.0301 (10)	0.0598 (13)	−0.0036 (7)	0.0138 (8)	−0.0111 (9)
N1	0.0596 (16)	0.0552 (17)	0.0381 (16)	−0.0014 (13)	0.0055 (13)	−0.0132 (13)
O11	0.128 (2)	0.0599 (16)	0.0584 (17)	−0.0334 (15)	−0.0091 (15)	−0.0057 (14)
O12	0.0604 (14)	0.0735 (16)	0.0505 (14)	0.0013 (12)	−0.0196 (12)	−0.0135 (12)
N2	0.0397 (13)	0.0312 (13)	0.0348 (13)	−0.0035 (11)	−0.0083 (11)	0.0009 (10)
O21	0.0453 (11)	0.0416 (11)	0.0513 (13)	0.0043 (9)	0.0009 (10)	−0.0124 (10)
O22	0.0651 (13)	0.0338 (11)	0.0642 (15)	−0.0196 (10)	0.0084 (11)	0.0007 (10)
C1	0.0258 (11)	0.0381 (14)	0.0207 (13)	−0.0041 (11)	0.0036 (9)	−0.0011 (11)
C2	0.0346 (14)	0.0388 (16)	0.0317 (16)	−0.0140 (12)	0.0027 (12)	0.0071 (13)
C3	0.0348 (14)	0.065 (2)	0.0256 (15)	−0.0094 (14)	0.0080 (12)	−0.0032 (14)
C11	0.0261 (12)	0.0291 (13)	0.0235 (13)	−0.0040 (10)	0.0019 (10)	−0.0018 (11)
C12	0.0246 (12)	0.0343 (14)	0.0264 (14)	−0.0007 (11)	0.0030 (10)	−0.0018 (12)
C13	0.0421 (14)	0.0374 (15)	0.0227 (14)	−0.0072 (12)	0.0056 (11)	−0.0009 (12)
C14	0.0368 (14)	0.0404 (16)	0.0272 (15)	−0.0054 (12)	−0.0058 (12)	0.0057 (12)
C15	0.0272 (13)	0.067 (2)	0.0388 (17)	0.0009 (13)	−0.0022 (13)	0.0059 (15)
C16	0.0283 (13)	0.0540 (17)	0.0328 (15)	−0.0025 (12)	0.0053 (11)	0.0000 (13)
C21	0.0257 (12)	0.0460 (16)	0.0258 (14)	0.0010 (12)	0.0008 (10)	0.0019 (13)
C22	0.0376 (14)	0.0480 (17)	0.0271 (15)	0.0060 (13)	0.0012 (11)	0.0040 (13)
C23	0.0468 (16)	0.0416 (16)	0.0324 (16)	0.0035 (13)	0.0087 (13)	0.0034 (13)
C24	0.0336 (14)	0.0450 (16)	0.0269 (15)	0.0042 (12)	0.0033 (11)	−0.0077 (13)
C25	0.0328 (14)	0.0489 (17)	0.0239 (14)	0.0120 (12)	0.0012 (11)	0.0002 (13)
C26	0.0316 (13)	0.0447 (16)	0.0264 (14)	0.0088 (12)	0.0034 (11)	0.0035 (13)
C31	0.0277 (13)	0.0294 (13)	0.0195 (13)	−0.0023 (10)	0.0058 (10)	−0.0029 (11)
C32	0.0299 (13)	0.0282 (13)	0.0277 (14)	0.0004 (11)	0.0030 (10)	0.0022 (11)
C33	0.0246 (12)	0.0334 (15)	0.0347 (15)	0.0044 (11)	0.0011 (11)	−0.0033 (12)
C34	0.0259 (12)	0.0273 (13)	0.0387 (15)	−0.0022 (10)	0.0097 (11)	−0.0089 (12)
C35	0.0339 (13)	0.0236 (13)	0.0382 (16)	−0.0018 (11)	0.0043 (11)	0.0004 (12)
C36	0.0280 (13)	0.0319 (14)	0.0321 (15)	0.0030 (11)	0.0022 (11)	−0.0012 (12)
C41	0.0246 (12)	0.0278 (14)	0.0312 (15)	0.0012 (10)	0.0003 (11)	−0.0016 (11)
C42	0.0253 (12)	0.0380 (15)	0.0287 (14)	−0.0012 (11)	0.0058 (10)	0.0022 (12)
C43	0.0286 (13)	0.0361 (15)	0.0312 (15)	−0.0083 (11)	0.0012 (11)	0.0084 (12)
C44	0.0317 (13)	0.0250 (13)	0.0272 (14)	−0.0004 (11)	−0.0039 (11)	0.0026 (11)
C45	0.0279 (13)	0.0343 (15)	0.0318 (15)	0.0000 (11)	0.0070 (11)	−0.0008 (12)
C46	0.0255 (12)	0.0312 (14)	0.0393 (16)	−0.0060 (10)	0.0091 (11)	0.0009 (12)

### Geometric parameters (Å, °)

**Table:**

F1—C2	1.342 (3)	C21—C26	1.388 (3)
F2—C2	1.338 (3)	C21—C22	1.391 (4)
F3—C2	1.336 (3)	C22—C23	1.388 (4)
F4—C3	1.339 (3)	C22—H22	0.9500
F5—C3	1.351 (3)	C23—C24	1.381 (4)
F6—C3	1.340 (3)	C23—H23	0.9500
O1—C21	1.374 (3)	C24—C25	1.379 (4)
O1—C14	1.408 (3)	C25—C26	1.376 (4)
O2—C41	1.389 (3)	C25—H25	0.9500
O2—C34	1.400 (3)	C26—H26	0.9500
N1—O12	1.224 (3)	C31—C32	1.393 (3)
N1—O11	1.226 (3)	C31—C36	1.400 (3)
N1—C24	1.463 (3)	C32—C33	1.391 (3)
N2—O22	1.230 (3)	C32—H32	0.9500
N2—O21	1.231 (3)	C33—C34	1.371 (3)
N2—C44	1.471 (3)	C33—H33	0.9500
C1—C3	1.533 (4)	C34—C35	1.395 (3)
C1—C31	1.547 (3)	C35—C36	1.387 (3)
C1—C11	1.550 (3)	C35—H35	0.9500
C1—C2	1.557 (4)	C36—H36	0.9500
C11—C12	1.393 (3)	C41—C46	1.381 (3)
C11—C16	1.394 (3)	C41—C42	1.383 (3)
C12—C13	1.391 (3)	C42—C43	1.380 (3)
C12—H12	0.9500	C42—H42	0.9500
C13—C14	1.372 (4)	C43—C44	1.380 (4)
C13—H13	0.9500	C43—H43	0.9500
C14—C15	1.365 (4)	C44—C45	1.381 (3)
C15—C16	1.395 (4)	C45—C46	1.380 (3)
C15—H15	0.9500	C45—H45	0.9500
C16—H16	0.9500	C46—H46	0.9500
			
C21—O1—C14	119.1 (2)	C21—C22—H22	120.4
C41—O2—C34	118.21 (18)	C24—C23—C22	119.0 (3)
O12—N1—O11	123.1 (3)	C24—C23—H23	120.5
O12—N1—C24	118.6 (3)	C22—C23—H23	120.5
O11—N1—C24	118.3 (3)	C25—C24—C23	122.0 (2)
O22—N2—O21	123.6 (2)	C25—C24—N1	119.0 (2)
O22—N2—C44	118.3 (2)	C23—C24—N1	119.0 (3)
O21—N2—C44	118.1 (2)	C26—C25—C24	119.2 (2)
C3—C1—C31	106.68 (19)	C26—C25—H25	120.4
C3—C1—C11	112.6 (2)	C24—C25—H25	120.4
C31—C1—C11	111.18 (19)	C25—C26—C21	119.7 (2)
C3—C1—C2	108.0 (2)	C25—C26—H26	120.1
C31—C1—C2	112.37 (19)	C21—C26—H26	120.1
C11—C1—C2	106.06 (19)	C32—C31—C36	118.1 (2)
F3—C2—F2	107.0 (2)	C32—C31—C1	122.9 (2)
F3—C2—F1	106.70 (19)	C36—C31—C1	118.8 (2)
F2—C2—F1	106.3 (2)	C33—C32—C31	121.3 (2)
F3—C2—C1	111.4 (2)	C33—C32—H32	119.3
F2—C2—C1	111.7 (2)	C31—C32—H32	119.3
F1—C2—C1	113.4 (2)	C34—C33—C32	119.2 (2)
F4—C3—F6	107.7 (2)	C34—C33—H33	120.4
F4—C3—F5	105.8 (2)	C32—C33—H33	120.4
F6—C3—F5	106.5 (2)	C33—C34—C35	121.4 (2)
F4—C3—C1	111.7 (2)	C33—C34—O2	117.9 (2)
F6—C3—C1	113.1 (2)	C35—C34—O2	120.5 (2)
F5—C3—C1	111.6 (2)	C36—C35—C34	118.7 (2)
C12—C11—C16	118.7 (2)	C36—C35—H35	120.6
C12—C11—C1	117.4 (2)	C34—C35—H35	120.6
C16—C11—C1	123.9 (2)	C35—C36—C31	121.3 (2)
C13—C12—C11	120.5 (2)	C35—C36—H36	119.4
C13—C12—H12	119.7	C31—C36—H36	119.4
C11—C12—H12	119.7	C46—C41—C42	121.8 (2)
C14—C13—C12	119.3 (3)	C46—C41—O2	122.4 (2)
C14—C13—H13	120.4	C42—C41—O2	115.7 (2)
C12—C13—H13	120.4	C43—C42—C41	119.3 (2)
C15—C14—C13	121.7 (2)	C43—C42—H42	120.3
C15—C14—O1	120.6 (2)	C41—C42—H42	120.3
C13—C14—O1	117.5 (2)	C42—C43—C44	118.4 (2)
C14—C15—C16	119.4 (2)	C42—C43—H43	120.8
C14—C15—H15	120.3	C44—C43—H43	120.8
C16—C15—H15	120.3	C43—C44—C45	122.6 (2)
C11—C16—C15	120.4 (3)	C43—C44—N2	119.4 (2)
C11—C16—H16	119.8	C45—C44—N2	118.0 (2)
C15—C16—H16	119.8	C46—C45—C44	118.7 (2)
O1—C21—C26	115.0 (2)	C46—C45—H45	120.7
O1—C21—C22	124.1 (2)	C44—C45—H45	120.7
C26—C21—C22	120.9 (2)	C45—C46—C41	119.1 (2)
C23—C22—C21	119.2 (2)	C45—C46—H46	120.4
C23—C22—H22	120.4	C41—C46—H46	120.4
			
C3—C1—C2—F3	−75.8 (3)	O12—N1—C24—C25	1.2 (4)
C31—C1—C2—F3	166.8 (2)	O11—N1—C24—C25	−178.3 (3)
C11—C1—C2—F3	45.1 (3)	O12—N1—C24—C23	−176.7 (3)
C3—C1—C2—F2	164.62 (19)	O11—N1—C24—C23	3.7 (4)
C31—C1—C2—F2	47.2 (3)	C23—C24—C25—C26	0.6 (4)
C11—C1—C2—F2	−74.5 (2)	N1—C24—C25—C26	−177.3 (2)
C3—C1—C2—F1	44.6 (3)	C24—C25—C26—C21	0.8 (4)
C31—C1—C2—F1	−72.8 (3)	O1—C21—C26—C25	−179.8 (2)
C11—C1—C2—F1	165.5 (2)	C22—C21—C26—C25	−1.5 (4)
C31—C1—C3—F4	−69.4 (2)	C3—C1—C31—C32	−89.6 (3)
C11—C1—C3—F4	52.8 (3)	C11—C1—C31—C32	147.3 (2)
C2—C1—C3—F4	169.59 (19)	C2—C1—C31—C32	28.6 (3)
C31—C1—C3—F6	168.9 (2)	C3—C1—C31—C36	85.6 (3)
C11—C1—C3—F6	−68.9 (3)	C11—C1—C31—C36	−37.5 (3)
C2—C1—C3—F6	47.9 (3)	C2—C1—C31—C36	−156.2 (2)
C31—C1—C3—F5	48.8 (3)	C36—C31—C32—C33	1.2 (4)
C11—C1—C3—F5	171.1 (2)	C1—C31—C32—C33	176.4 (2)
C2—C1—C3—F5	−72.2 (3)	C31—C32—C33—C34	−0.9 (4)
C3—C1—C11—C12	−168.0 (2)	C32—C33—C34—C35	0.6 (4)
C31—C1—C11—C12	−48.3 (3)	C32—C33—C34—O2	176.3 (2)
C2—C1—C11—C12	74.1 (3)	C41—O2—C34—C33	125.7 (2)
C3—C1—C11—C16	13.8 (3)	C41—O2—C34—C35	−58.6 (3)
C31—C1—C11—C16	133.5 (2)	C33—C34—C35—C36	−0.7 (4)
C2—C1—C11—C16	−104.1 (3)	O2—C34—C35—C36	−176.2 (2)
C16—C11—C12—C13	−0.7 (4)	C34—C35—C36—C31	1.0 (4)
C1—C11—C12—C13	−179.0 (2)	C32—C31—C36—C35	−1.2 (4)
C11—C12—C13—C14	−0.1 (4)	C1—C31—C36—C35	−176.7 (2)
C12—C13—C14—C15	0.7 (4)	C34—O2—C41—C46	−24.7 (3)
C12—C13—C14—O1	−175.2 (2)	C34—O2—C41—C42	157.6 (2)
C21—O1—C14—C15	74.2 (3)	C46—C41—C42—C43	1.5 (4)
C21—O1—C14—C13	−109.9 (3)	O2—C41—C42—C43	179.2 (2)
C13—C14—C15—C16	−0.6 (4)	C41—C42—C43—C44	0.0 (3)
O1—C14—C15—C16	175.2 (2)	C42—C43—C44—C45	−0.8 (4)
C12—C11—C16—C15	0.9 (4)	C42—C43—C44—N2	177.0 (2)
C1—C11—C16—C15	179.1 (2)	O22—N2—C44—C43	5.5 (3)
C14—C15—C16—C11	−0.3 (4)	O21—N2—C44—C43	−173.5 (2)
C14—O1—C21—C26	−171.1 (2)	O22—N2—C44—C45	−176.7 (2)
C14—O1—C21—C22	10.6 (4)	O21—N2—C44—C45	4.4 (3)
O1—C21—C22—C23	179.0 (2)	C43—C44—C45—C46	0.1 (4)
C26—C21—C22—C23	0.8 (4)	N2—C44—C45—C46	−177.7 (2)
C21—C22—C23—C24	0.5 (4)	C44—C45—C46—C41	1.4 (3)
C22—C23—C24—C25	−1.2 (4)	C42—C41—C46—C45	−2.2 (4)
C22—C23—C24—N1	176.7 (2)	O2—C41—C46—C45	−179.8 (2)

### Hydrogen-bond geometry (Å, °)

**Table:**

*D*—H···*A*	*D*—H	H···*A*	*D*···*A*	*D*—H···*A*
C46—H46···Cg1^i^	0.95	3.04	3.710	129

Symmetry codes: (i) *x*, −*y*+1/2, *z*+1/2.
